# Supplementary material for: The Role of Psychological Flexibility and Psychological Factors in Chronic Pelvic Pain Among Women: A Correlational Study
Source: Healthcare (Basel). 2025 Jul 15;13(14):1697. doi: 10.3390/healthcare13141697 (PMC12295592; doi:10.3390/healthcare13141697)
Supplement: Supplementary file 1 [file healthcare-13-01697-s001.zip › healthcare-3678642-supplementary.pdf]

Supplementary Materials: The Role of Psychological Flexibility and Psychological Factors in Chronic Pelvic Pain Among Women: A Correlational Study

**Table S1: Descriptive Statistics**

| | Età | Anxiety | Depression | Stress | Acceptance | Contact w/present moment | Self as context | Defusion | Values | Actions | Psycological Flexibility | Experiential avoidance |
|---|---|---|---|---|---|---|---|---|---|---|---|---|
| N | 114 | 115 | 115 | 115 | 74 | 74 | 74 | 74 | 74 | 74 | 74 | 74 |
| Mancanti | 1 | 0 | 0 | 0 | 41 | 41 | 41 | 41 | 41 | 41 | 41 | 41 |
| Media | 35.1 | 15.0 | 20.1 | 22.1 | 3.38 | 3.93 | 3.35 | 2.93 | 3.79 | 3.67 | 3.51 | 3.33 |
| Mediana | 32.0 | 14 | 18 | 22 | 3.40 | 4.00 | 3.00 | 2.90 | 3.80 | 3.60 | 3.45 | 3.20 |
| Deviazione standard | 20.0 | 7.82 | 8.80 | 6.95 | 0.833 | 0.998 | 1.04 | 0.917 | 1.09 | 1.06 | 0.761 | 1.08 |
| Minimo | 18 | 0 | 4 | 4 | 1.60 | 2.00 | 1.20 | 1.00 | 1.40 | 1.60 | 2.10 | 1.00 |
| Massimo | 227 | 34 | 40 | 36 | 5.60 | 6.00 | 5.60 | 5.00 | 6.00 | 6.00 | 5.17 | 6.00 |
| Asimmetria | 7.99 | 0.426 | 0.477 | -0.149 | 0.292 | 0.140 | 0.326 | 0.322 | -0.134 | 0.283 | 0.274 | 0.328 |
| Errore std. asimmetria | 0.226 | 0.226 | 0.226 | 0.226 | 0.279 | 0.279 | 0.279 | 0.279 | 0.279 | 0.279 | 0.279 | 0.279 |
| Curtosi | 76.5 | -0.409 | -0.624 | -0.584 | -0.195 | -0.390 | -0.700 | -0.265 | -0.798 | -0.503 | -0.753 | -0.371 |
| Err. Std. di Curtosi | 0.449 | 0.447 | 0.447 | 0.447 | 0.552 | 0.552 | 0.552 | 0.552 | 0.552 | 0.552 | 0.552 | 0.552 |
| Shapiro-Wilk W | 0.401 | 0.967 | 0.956 | 0.979 | 0.985 | 0.980 | 0.966 | 0.978 | 0.977 | 0.978 | 0.975 | 0.976 |
| Shapiro-Wilk p | <?.001 | 0.006 | <?.001 | 0.074 | 0.538 | 0.292 | 0.042 | 0.236 | 0.191 | 0.220 | 0.143 | 0.170 |

| | Contact_Inf | Self as content | Cognitive Fusion | Values_Inf | Actions_Inf | Psychological Inflexibility | Pain interference | Pain intensity | NRS | SF12_PCS | SF12_MCS |
|---|---|---|---|---|---|---|---|---|---|---|---|
| N | 74 | 74 | 74 | 74 | 74 | 74 | 60 | 64 | 62 | 114 | 114 |
| Mancanti | 41 | 41 | 41 | 41 | 41 | 41 | 55 | 51 | 53 | 1 | 1 |
| Media | 3.01 | 2.96 | 3.24 | 2.76 | 2.64 | 2.99 | 5.72 | 4.74 | 5.15 | 39.8 | 38.5 |
| Mediana | 3.00 | 3.00 | 3.20 | 2.40 | 2.40 | 2.97 | 5.86 | 5.13 | 6.00 | 38.3 | 37.0 |
| Deviazione standard | 1.16 | 1.27 | 1.30 | 1.26 | 1.20 | 0.846 | 2.15 | 1.97 | 2.64 | 11.2 | 11.6 |
| Minimo | 1.00 | 1.00 | 1.00 | 1.00 | 1.00 | 1.03 | 0.143 | 0.00 | 0 | 17.4 | 13.2 |
| Massimo | 6.00 | 6.00 | 5.60 | 6.00 | 6.00 | 4.93 | 9.71 | 9.25 | 9 | 64.0 | 61.6 |
| Asimmetria | 0.593 | 0.330 | 0.169 | 0.635 | 0.702 | 0.201 | -0.370 | -0.384 | -0.664 | 0.329 | 0.143 |
| Errore std. asimmetria | 0.279 | 0.279 | 0.279 | 0.279 | 0.279 | 0.279 | 0.309 | 0.299 | 0.304 | 0.226 | 0.226 |
| Curtosi | 0.00883 | -0.641 | -0.928 | -0.630 | 0.0961 | -0.231 | -0.175 | 0.0593 | -0.653 | -0.785 | -0.670 |
| Err. Std. di Curtosi | 0.552 | 0.552 | 0.552 | 0.552 | 0.552 | 0.552 | 0.608 | 0.590 | 0.599 | 0.449 | 0.449 |
| Shapiro-Wilk W | 0.964 | 0.969 | 0.961 | 0.929 | 0.946 | 0.989 | 0.981 | 0.972 | 0.903 | 0.966 | 0.976 |
| Shapiro-Wilk p | 0.033 | 0.066 | 0.024 | <?.001 | 0.003 | 0.755 | 0.494 | 0.147 | <?.001 | 0.005 | 0.036 |

**Table S2: Correlational Analysis between Psychological Distress and Pain**

| | | Anxiety | Depression | Stress | Pain intensity | Pain interference | NRS |
|---|---|---|---|---|---|---|---|
| Anxiety | Rho di Spearman | — | | | | | |
| | valore p | — | | | | | |
| Depression | Rho di Spearman | 0.568 *** | — | | | | |
| | valore p | < .001 | — | | | | |
| Stress | Rho di Spearman | 0.667 *** | 0.697 *** | — | | | |
| | valore p | < .001 | < .001 | — | | | |
| Pain intensity | Rho di Spearman | 0.111 | 0.074 | 0.202 | — | | |
| | valore p | 0.383 | 0.561 | 0.109 | — | | |
| Pain interference | Rho di Spearman | 0.324 * | 0.302 * | 0.481 *** | 0.644 *** | — | |
| | valore p | 0.011 | 0.019 | < .001 | < .001 | — | |
| NRS | Rho di Spearman | 0.211 | 0.250 | 0.321 * | 0.755 *** | 0.462 *** | — |
| | valore p | 0.100 | 0.050 | 0.011 | < .001 | < .001 | — |

Nota. * p < .05, ** p < .01, *** p < .001

Supplementary Materials:

**Table S3: Correlation Analysis between Psychological Flexibility and Pain**

| | | Acceptance | Contact w/present moment | Self as context | Defusion | Values | Actions | Psycological Flexibility | Pain interference | Pain intensity | NRS |
|---|---|---|---|---|---|---|---|---|---|---|---|
| Acceptance | Rho di Spearman | — | | | | | | | -0.227 | | |
| | valore p | — | | | | | | | 0.081 | | |
| Contact w/present moment | Rho di Spearman | 0.483 *** | — | | | | | | | | |
| | valore p | < .001 | — | | | | | | | | |
| Self as context | Rho di Spearman | 0.593 *** | 0.376 *** | — | | | | | -0.302 * | | |
| | valore p | < .001 | < .001 | — | | | | | 0.019 | | |
| Defusion | Rho di Spearman | 0.581 *** | 0.189 | 0.708 *** | — | | | | -0.361 ** | | |
| | valore p | < .001 | 0.107 | < .001 | — | | | | 0.005 | | |
| Values | Rho di Spearman | 0.473 *** | 0.483 *** | 0.704 *** | 0.496 *** | — | | | -0.257 * | | |
| | valore p | < .001 | < .001 | < .001 | < .001 | — | | | 0.048 | | |
| Actions | Rho di Spearman | 0.382 *** | 0.310 ** | 0.606 *** | 0.466 *** | 0.773 *** | — | | -0.253 | | |
| | valore p | < .001 | 0.007 | < .001 | < .001 | < .001 | — | | 0.051 | | |
| Psycological Flexibility | Rho di Spearman | 0.716 *** | 0.615 *** | 0.869 *** | 0.705 *** | 0.881 *** | 0.787 *** | — | -0.299 * | | |
| | valore p | < .001 | < .001 | < .001 | < .001 | < .001 | < .001 | — | 0.020 | | |
| Pain interference | Rho di Spearman | -0.227 | 0.038 | -0.302 * | -0.361 ** | -0.257 * | -0.253 | -0.299 * | — | | |
| | valore p | 0.081 | 0.771 | 0.019 | 0.005 | 0.048 | 0.051 | 0.020 | — | | |

**Table S3: Correlation Analysis between Psychological Flexibility and Pain**

| | | Acceptance | Contact w/present moment | Self as context | Defusion | Values | Actions | Psycological Flexibility | Pain interference | Pain intensity | NRS |
|---|---|---|---|---|---|---|---|---|---|---|---|
| Pain intensity | Rho di Spearman | -0.068 | -0.095 | -0.141 | -0.133 | -0.152 | -0.135 | -0.156 | 0.644 *** | — | |
| | valore p | 0.594 | 0.455 | 0.268 | 0.296 | 0.230 | 0.286 | 0.219 | < .001 | — | |
| NRS | Rho di Spearman | 0.010 | 0.006 | -0.121 | -0.190 | -0.158 | -0.145 | -0.136 | 0.462 *** | 0.755 *** | — |
| | valore p | 0.936 | 0.961 | 0.347 | 0.139 | 0.220 | 0.260 | 0.292 | < .001 | < .001 | — |

Nota. * p < .05, ** p < .01, *** p < .001

**Table S4: Correlational analysis between Psychological Inflexibility and Pain**

| | | Experiential avoidance | Contact_Inf | Self as content | Cognitive Fusion | Values_Inf | Actions_Inf | Psychological Inflexibility | Pain interference | Pain intensity | NRS |
|---|---|---|---|---|---|---|---|---|---|---|---|
| Experiential avoidance | Rho di Spearman | — | | | | | | | | | |
| | gdl | — | | | | | | | | | |
| | valore p | — | | | | | | | | | |
| Contact_Inf | Rho di Spearman | 0.123 | — | | | | | | | | |
| | gdl | 72 | — | | | | | | | | |
| | valore p | 0.298 | — | | | | | | | | |
| Self as content | Rho di Spearman | 0.218 | 0.321 ** | — | | | | | | | |
| | gdl | 72 | 72 | — | | | | | | | |
| | valore p | 0.062 | 0.005 | — | | | | | | | |
| Cognitive Fusion | Rho di Spearman | 0.039 | 0.375 *** | 0.627 *** | — | | | | | | |
| | gdl | 72 | 72 | 72 | — | | | | | | |
| | valore p | 0.744 | < .001 | < .001 | — | | | | | | |
| Values_Inf | Rho di Spearman | -0.003 | 0.496 *** | 0.283 * | 0.566 *** | — | | | | | |
| | gdl | 72 | 72 | 72 | 72 | — | | | | | |
| | valore p | 0.978 | < .001 | 0.014 | < .001 | — | | | | | |
| Actions_Inf | Rho di Spearman | -0.022 | 0.594 *** | 0.518 *** | 0.705 *** | 0.681 *** | — | | | | |
| | gdl | 72 | 72 | 72 | 72 | 72 | — | | | | |
| | valore p | 0.850 | < .001 | < .001 | < .001 | < .001 | — | | | | |
| Psychological Inflexibility | Rho di Spearman | 0.250 * | 0.661 *** | 0.727 *** | 0.826 *** | 0.725 *** | 0.844 *** | — | | | |

**Table S4: Correlational analysis between Psychological Inflexibility and Pain**

| | | Experiential avoidance | Contact_Inf | Self as content | Cognitive Fusion | Values_Inf | Actions_Inf | Psychological Inflexibility | Pain interference | Pain intensity | NRS |
|---|---|---|---|---|---|---|---|---|---|---|---|
| | gdl | 72 | 72 | 72 | 72 | 72 | 72 | — | | | |
| | valore p | 0.032 | < .001 | < .001 | < .001 | < .001 | < .001 | — | | | |
| Pain interference | Rho di Spearman | -0.137 | 0.127 | 0.020 | 0.247 | 0.253 | 0.245 | 0.224 | — | | |
| | gdl | 58 | 58 | 58 | 58 | 58 | 58 | 58 | — | | |
| | valore p | 0.298 | 0.334 | 0.881 | 0.057 | 0.051 | 0.060 | 0.085 | — | | |
| Pain intensity | Rho di Spearman | -0.032 | 0.082 | 0.002 | 0.184 | 0.208 | 0.112 | 0.102 | 0.644 *** | — | |
| | gdl | 62 | 62 | 62 | 62 | 62 | 62 | 62 | 58 | — | |
| | valore p | 0.799 | 0.522 | 0.986 | 0.146 | 0.099 | 0.380 | 0.421 | < .001 | — | |
| NRS | Rho di Spearman | 0.043 | 0.066 | 0.165 | 0.332 ** | 0.281 * | 0.248 | 0.242 | 0.462 *** | 0.755 *** | — |
| | gdl | 60 | 60 | 60 | 60 | 60 | 60 | 60 | 58 | 60 | — |
| | valore p | 0.743 | 0.609 | 0.200 | 0.008 | 0.027 | 0.052 | 0.058 | < .001 | < .001 | — |

Nota. * p < .05, ** p < .01, *** p < .001

**Table S5: Correlational Analysis between Psychological Inflexibility and Mental quality of life**

| | | Experiential avoidance | Contact_Inf | Self as content | Cognitive Fusion | Values_Inf | Actions_Inf | Psychological Inflexibility | SF12_MCS |
|---|---|---|---|---|---|---|---|---|---|
| Experiential avoidance | Rho di Spearman | — | | | | | | | |
| | gdl | — | | | | | | | |
| | valore p | — | | | | | | | |
| Contact_Inf | Rho di Spearman | 0.123 | — | | | | | | |
| | gdl | 72 | — | | | | | | |
| | valore p | 0.298 | — | | | | | | |
| Self as content | Rho di Spearman | 0.218 | 0.321 ** | — | | | | | |
| | gdl | 72 | 72 | — | | | | | |
| | valore p | 0.062 | 0.005 | — | | | | | |
| Cognitive Fusion | Rho di Spearman | 0.039 | 0.375 *** | 0.627 *** | — | | | | |
| | gdl | 72 | 72 | 72 | — | | | | |
| | valore p | 0.744 | < .001 | < .001 | — | | | | |
| Values_Inf | Rho di Spearman | -0.003 | 0.496 *** | 0.283 * | 0.566 *** | — | | | |
| | gdl | 72 | 72 | 72 | 72 | — | | | |
| | valore p | 0.978 | < .001 | 0.014 | < .001 | — | | | |
| Actions_Inf | Rho di Spearman | -0.022 | 0.594 *** | 0.518 *** | 0.705 *** | 0.681 *** | — | | |
| | gdl | 72 | 72 | 72 | 72 | 72 | — | | |
| | valore p | 0.850 | < .001 | < .001 | < .001 | < .001 | — | | |
| Psychological Inflexibility | Rho di Spearman | 0.250 * | 0.661 *** | 0.727 *** | 0.826 *** | 0.725 *** | 0.844 *** | — | |

**Table S5: Correlational Analysis between Psychological Inflexibility and Mental quality of life**

| | | Experiential avoidance | Contact_Inf | Self as content | Cognitive Fusion | Values_Inf | Actions_Inf | Psychological Inflexibility | SF12_MCS |
|---|---|---|---|---|---|---|---|---|---|
| | gdl | 72 | 72 | 72 | 72 | 72 | 72 | — | |
| | valore p | 0.032 | < .001 | < .001 | < .001 | < .001 | < .001 | — | |
| SF12_MCS | Rho di Spearman | 0.153 | 0.003 | 0.332 ** | 0.204 | 0.058 | 0.150 | 0.239 * | — |
| | gdl | 71 | 71 | 71 | 71 | 71 | 71 | 71 | — |
| | valore p | 0.197 | 0.979 | 0.004 | 0.083 | 0.628 | 0.205 | 0.042 | — |

Nota. * p < .05, ** p < .01, *** p < .001

**Table S6: Correlational analysis between Psychological inflexibility and Physical quality of life**

| | | Experiential avoidance | Contact_Inf | Self as content | Cognitive Fusion | Values_Inf | Actions_Inf | Psychological Inflexibility | SF12_PCS |
|---|---|---|---|---|---|---|---|---|---|
| Experiential avoidance | Rho di Spearman | — | | | | | | | |
| | gdl | — | | | | | | | |
| | valore p | — | | | | | | | |
| Contact_Inf | Rho di Spearman | 0.123 | — | | | | | | |
| | gdl | 72 | — | | | | | | |
| | valore p | 0.298 | — | | | | | | |
| Self as content | Rho di Spearman | 0.218 | 0.321 ** | — | | | | | |
| | gdl | 72 | 72 | — | | | | | |
| | valore p | 0.062 | 0.005 | — | | | | | |
| Cognitive Fusion | Rho di Spearman | 0.039 | 0.375 *** | 0.627 *** | — | | | | |
| | gdl | 72 | 72 | 72 | — | | | | |
| | valore p | 0.744 | < .001 | < .001 | — | | | | |
| Values_Inf | Rho di Spearman | -0.003 | 0.496 *** | 0.283 * | 0.566 *** | — | | | |
| | gdl | 72 | 72 | 72 | 72 | — | | | |
| | valore p | 0.978 | < .001 | 0.014 | < .001 | — | | | |
| Actions_Inf | Rho di Spearman | -0.022 | 0.594 *** | 0.518 *** | 0.705 *** | 0.681 *** | — | | |
| | gdl | 72 | 72 | 72 | 72 | 72 | — | | |
| | valore p | 0.850 | < .001 | < .001 | < .001 | < .001 | — | | |
| Psychological Inflexibility | Rho di Spearman | 0.250 * | 0.661 *** | 0.727 *** | 0.826 *** | 0.725 *** | 0.844 *** | — | |

**Table S6: Correlational analysis between Psychological inflexibility and Physical quality of life**

| | | Experiential avoidance | Contact_Inf | Self as content | Cognitive Fusion | Values_Inf | Actions_Inf | Psychological Inflexibility | SF12_PCS |
|---|---|---|---|---|---|---|---|---|---|
| | gdl | 72 | 72 | 72 | 72 | 72 | 72 | — | |
| | valore p | 0.032 | < .001 | < .001 | < .001 | < .001 | < .001 | — | |
| SF12_PCS | Rho di Spearman | 0.273 * | -0.019 | 0.132 | -0.115 | -0.236 * | -0.085 | -0.015 | — |
| | gdl | 71 | 71 | 71 | 71 | 71 | 71 | 71 | — |
| | valore p | 0.019 | 0.871 | 0.266 | 0.332 | 0.045 | 0.475 | 0.900 | — |

Nota. * p < .05, ** p < .01, *** p < .001

**Table S7: Correlational analysis between Psychological inflexibility and psychological distress**

| | | Anxiety | Depression | Stress | Experiential avoidance | Contact_Inf | Self as content | Cognitive Fusion | Values_Inf | Actions_Inf | Psychological Inflexibility |
|---|---|---|---|---|---|---|---|---|---|---|---|
| Anxiety | Rho di Spearman | — | | | | | | | | | |
| | gdl | — | | | | | | | | | |
| | valore p | — | | | | | | | | | |
| Depression | Rho di Spearman | 0.568 *** | — | | | | | | | | |
| | gdl | 113 | — | | | | | | | | |
| | valore p | < .001 | — | | | | | | | | |
| Stress | Rho di Spearman | 0.667 *** | 0.697 *** | — | | | | | | | |
| | gdl | 113 | 113 | — | | | | | | | |
| | valore p | < .001 | < .001 | — | | | | | | | |
| Experiential avoidance | Rho di Spearman | 0.034 | 0.077 | -0.003 | — | | | | | | |
| | gdl | 72 | 72 | 72 | — | | | | | | |
| | valore p | 0.775 | 0.513 | 0.980 | — | | | | | | |
| Contact_Inf | Rho di Spearman | 0.351 ** | 0.413 *** | 0.397 *** | 0.123 | — | | | | | |
| | gdl | 72 | 72 | 72 | 72 | — | | | | | |
| | valore p | 0.002 | < .001 | < .001 | 0.298 | — | | | | | |
| Self as content | Rho di Spearman | 0.198 | 0.332 ** | 0.152 | 0.218 | 0.321 ** | — | | | | |
| | gdl | 72 | 72 | 72 | 72 | 72 | — | | | | |
| | valore p | 0.091 | 0.004 | 0.197 | 0.062 | 0.005 | — | | | | |
| Cognitive Fusion | Rho di Spearman | 0.317 ** | 0.506 *** | 0.379 *** | 0.039 | 0.375 *** | 0.627 *** | — | | | |

**Table S7: Correlational analysis between Psychological inflexibility and psychological distress**

| | | Anxiety | Depression | Stress | Experiential avoidance | Contact_Inf | Self as content | Cognitive Fusion | Values_Inf | Actions_Inf | Psychological Inflexibility |
|---|---|---|---|---|---|---|---|---|---|---|---|
| | gdl | 72 | 72 | 72 | 72 | 72 | 72 | — | | | |
| | valore p | 0.006 | < .001 | < .001 | 0.744 | < .001 | < .001 | — | | | |
| Values_Inf | Rho di Spearman | 0.257 * | 0.430 *** | 0.419 *** | -0.003 | 0.496 *** | 0.283 * | 0.566 *** | — | | |
| | gdl | 72 | 72 | 72 | 72 | 72 | 72 | 72 | — | | |
| | valore p | 0.027 | < .001 | < .001 | 0.978 | < .001 | 0.014 | < .001 | — | | |
| Actions_Inf | Rho di Spearman | 0.420 *** | 0.605 *** | 0.520 *** | -0.022 | 0.594 *** | 0.518 *** | 0.705 *** | 0.681 *** | — | |
| | gdl | 72 | 72 | 72 | 72 | 72 | 72 | 72 | 72 | — | |
| | valore p | < .001 | < .001 | < .001 | 0.850 | < .001 | < .001 | < .001 | < .001 | — | |
| Psychological Inflexibility | Rho di Spearman | 0.396 *** | 0.579 *** | 0.470 *** | 0.250 * | 0.661 *** | 0.727 *** | 0.826 *** | 0.725 *** | 0.844 *** | — |
| | gdl | 72 | 72 | 72 | 72 | 72 | 72 | 72 | 72 | 72 | — |
| | valore p | < .001 | < .001 | < .001 | 0.032 | < .001 | < .001 | < .001 | < .001 | < .001 | — |

Nota. * p < .05, ** p < .01, *** p < .001

**Table S8: Correlational analysis between Psychological Flexibility and psychological distress**

| | | Anxiety | Depression | Stress | Acceptance | Contact w/present moment | Self as context | Defusion | Values | Actions | Psycological Flexibility |
|---|---|---|---|---|---|---|---|---|---|---|---|
| Anxiety | Rho di Spearman | — | | | | | | | | | |
| | gdl | — | | | | | | | | | |
| | valore p | — | | | | | | | | | |
| Depression | Rho di Spearman | 0.568 *** | — | | | | | | | | |
| | gdl | 113 | — | | | | | | | | |
| | valore p | < .001 | — | | | | | | | | |
| Stress | Rho di Spearman | 0.667 *** | 0.697 *** | — | | | | | | | |
| | gdl | 113 | 113 | — | | | | | | | |
| | valore p | < .001 | < .001 | — | | | | | | | |
| Acceptance | Rho di Spearman | 0.113 | -0.042 | 0.020 | — | | | | | | |
| | gdl | 72 | 72 | 72 | — | | | | | | |
| | valore p | 0.339 | 0.722 | 0.868 | — | | | | | | |
| Contact w/present moment | Rho di Spearman | -0.056 | 0.005 | 0.036 | 0.483 *** | — | | | | | |
| | gdl | 72 | 72 | 72 | 72 | — | | | | | |
| | valore p | 0.637 | 0.969 | 0.758 | < .001 | — | | | | | |
| Self as context | Rho di Spearman | -0.091 | -0.155 | -0.273 * | 0.593 *** | 0.376 *** | — | | | | |
| | gdl | 72 | 72 | 72 | 72 | 72 | — | | | | |

**Table S8: Correlational analysis between Psychological Flexibility and psychological distress**

| | | Anxiety | Depression | Stress | Acceptance | Contact w/present moment | Self as context | Defusion | Values | Actions | Psycological Flexibility |
|---|---|---|---|---|---|---|---|---|---|---|---|
| | valore p | 0.443 | 0.187 | 0.019 | < .001 | < .001 | — | | | | |
| Defusion | Rho di Spearman | - 0.129 | -0.147 | - 0.297 * | 0.581 *** | 0.189 | 0.708 *** | — | | | |
| | gdl | 72 | 72 | 72 | 72 | 72 | 72 | — | | | |
| | valore p | 0.273 | 0.213 | 0.010 | < .001 | 0.107 | < .001 | — | | | |
| Values | Rho di Spearman | - 0.006 | -0.114 | - 0.141 | 0.473 *** | 0.483 *** | 0.704 *** | 0.496 *** | — | | |
| | gdl | 72 | 72 | 72 | 72 | 72 | 72 | 72 | — | | |
| | valore p | 0.958 | 0.332 | 0.232 | < .001 | < .001 | < .001 | < .001 | — | | |
| Actions | Rho di Spearman | - 0.155 | -0.212 | - 0.216 | 0.382 *** | 0.310 ** | 0.606 *** | 0.466 *** | 0.773 *** | — | |
| | gdl | 72 | 72 | 72 | 72 | 72 | 72 | 72 | 72 | — | |
| | valore p | 0.189 | 0.070 | 0.065 | < .001 | 0.007 | < .001 | < .001 | < .001 | — | |
| Psycological Flexibility | Rho di Spearman | - 0.083 | -0.155 | - 0.197 | 0.716 *** | 0.615 *** | 0.869 *** | 0.705 *** | 0.881 *** | 0.787 *** | — |
| | gdl | 72 | 72 | 72 | 72 | 72 | 72 | 72 | 72 | 72 | — |
| | valore p | 0.484 | 0.187 | 0.093 | < .001 | < .001 | < .001 | < .001 | < .001 | < .001 | — |

Nota. * p < .05, ** p < .01, *** p < .001
